# Challenges and opportunities in bringing non-HLA antibody testing for post-transplant monitoring

**DOI:** 10.3389/frtra.2025.1594241

**Published:** 2025-06-05

**Authors:** Mary Carmelle Philogene, Inna Tchoukina, Idoia Gimferrer

**Affiliations:** ^1^ Histocompatibility and Immunogenetics Laboratory, Virginia Commonwealth University, Richmond, VA, United States; ^2^ Heart Failure/ Transplantation, Division of Cardiology, Virginia Commonwealth University, Richmond, VA, United States; ^3^ Immunogenetics/HLA Laboratory at Bloodworks NorthWest, Seattle, WA, United States

**Keywords:** luminex testing, HLA antibodies, non-HLA antibodies, AT1RAbs, assay standardization

## Abstract

Evidence for the contribution of non-HLA antibodies on long-term allograft outcome was suggested in early studies by Paul Terasaki and colleagues who showed worse 10-year allograft outcome in HLA identical kidney transplant recipients with a positive panel reactive antibody (PRA) as determined by the micro cytotoxicity assay, in which cells express other targets beside HLA. More recent reports have shown worse graft outcome when antibodies against non-HLA antigens were detected with HLA-donor specific antibodies (HLA-DSA), and even suggest that non-HLA antibodies may serve as precursor to development of HLA antibodies. Unfortunately, the recent studies lack reproducibility, which then leads to skepticism as to the relevance of non-HLA antibody in transplantation outcome. Consequently, routine testing for non-HLA antibody along with monitoring of HLA-DSA as part of a post-transplant immune surveillance protocol is not standard practice. The Sensitization in Transplantation: Assessment of Risk (STAR) workgroup summarized the current literature on this topic, citing differences in cohort characteristics, variability in study design, selection of sample and timepoints for testing and variability in the assays used to detect non-HLA antibodies, as reasons that impact the accurate assessment on the relevance of non-HLA antibodies. However, correlation between test results and outcome can only be determined if the assay in question is detecting the correct analyte. Therefore, here we will make the case for a plan that requires a systematic validation of high-throughput bead-based assays, to include appropriate sequence selection for non-HLA antigenic targets and quality control metrics as a first step to solving this puzzle.

## Introduction: the clinical relevance of non-HLA antibody in transplantation

Despite progress in long-term transplant allograft survival, alloimmune injury remains problematic and reduces the longevity of an allograft (1). Routine monitoring for presence of donor specific HLA antibody (HLA-DSA), using assays with increased sensitivity and specificity, at various intervals post-transplantation helps mitigate the impact of HLA antibody on graft survival (2). However, antibody mediated injury has been reported on biopsy examination in patients with no detectable HLA-DSA (3). Immune response against targets other than HLA antigens has been associated with rejection in kidney (4), heart (5, 6), lung (7) and liver transplantation (8). Evidence for the contribution of these antibodies, coined “non-HLA antibodies”, on long-term allograft outcome was suggested in early studies by Paul Terasaki and colleagues (9). The study concluded that 30–40% of transplant recipients presented with antibody mediated rejection (AMR) on biopsy with no detectable HLA-DSA (10). Using a conservative mean fluorescence intensity (MFI) cutoff of ≥500 to report HLA-DSA in a kidney transplant cohort, Senev et al. demonstrated that 59% of cases that met the BANFF histological criteria for AMR did not have circulating HLA-DSA (11). Importantly, the study did not include testing for non-HLA antibody. Reports have shown worse graft outcome when antibodies against non-HLA antigens were detected with HLA-DSA (12), and even suggest that non-HLA antibodies may serve as precursor to development of HLA antibodies (13–15). In contrast, a prospective cohort study of 1,845 kidney transplants showed an increase in allograft loss in patients with post-transplant angiotensin II type 1 receptor antibodies (AT1RAb) and no HLA-DSA (16) and this effect was dose dependent. Furthermore, in cases where rejection was due to non-HLA antibody, treatment, consisting primarily of plasmapheresis to remove circulating antibodies, resolved the rejection (14 17–18). These studies underscore the relevance of non-HLA antibody in transplantation outcome. A systematic review of the current literature by the STAR workgroup identified shortcomings in the existing reports and made recommendations (19). Notably, the need for validation of high-throughput bead-based assays, to include appropriate quality control metrics is an important first step.

### Addressing the problem of assay specificity

Antibodies in patient sera are polyclonal and may bind to unique sequences, epitopes, and even post-translational modification features of a protein. However, to be activating or functional, the antibody must interact with the appropriate target. This was illustrated in the case of AT1RAb. Epitope analysis identified specific sequences AFHYESQ and ENTINIT, within the second extracellular loop of AT1R, as targets for activating antibodies (20). Mechanistic studies demonstrated that antibody binding to these sequences would “turn on” the receptor. The interaction between the antibody and the target sequences results in activation of downstream pathways leading to development of pro-inflammatory events (20). These sequences are also the target of therapeutic agents. Addition of Losartan, an angiotensin receptor blocker, reduced the activation of the receptor (20). The commercially available enzyme-linked immunosorbent assay (ELISA-AT1RAb) has undergone some scrutiny due to a lack of specificity. Using this assay, AT1RAbs have been detected in healthy individuals and transplant recipients with no graft dysfunction (21). In the ELISA, the entire AT1R protein, in its native conformation is overexpressed on Chinese hamster ovary (CHO) cells (22). Therefore, antibody detected in this assay may be directed against AT1R epitopes other than those defined in the mechanistic studies or against other proteins expressed on CHO cells or xenoantigens (23). Other ELISAs, not commercially available, and displaying the peptides described as target of the functional AT1RAbs, rather than the entire protein, have shown higher correlation with AT1RAb in patients with preeclampsia (24), hypertension and inflammation (25), supporting the pathogenesis of the activating AT1RAbs.

### Commercial luminex non-HLA antibody panels

To be used in a clinical setting, a non-HLA antibody assay must show accuracy, precision, reproducibility, should utilize samples that are easily acquired from the recipient, have a rapid turnaround time, and must include acceptable and reliable controls. Luminex based assays, widely used for detection of HLA antibodies, meet these requirements. Two multiplex bead-based non-HLA assays have been on the market for a while, but have not been fully incorporated into routine testing in clinical laboratories even though a few studies have reported data for either of these assays (26, 27).

**Figure 1:**
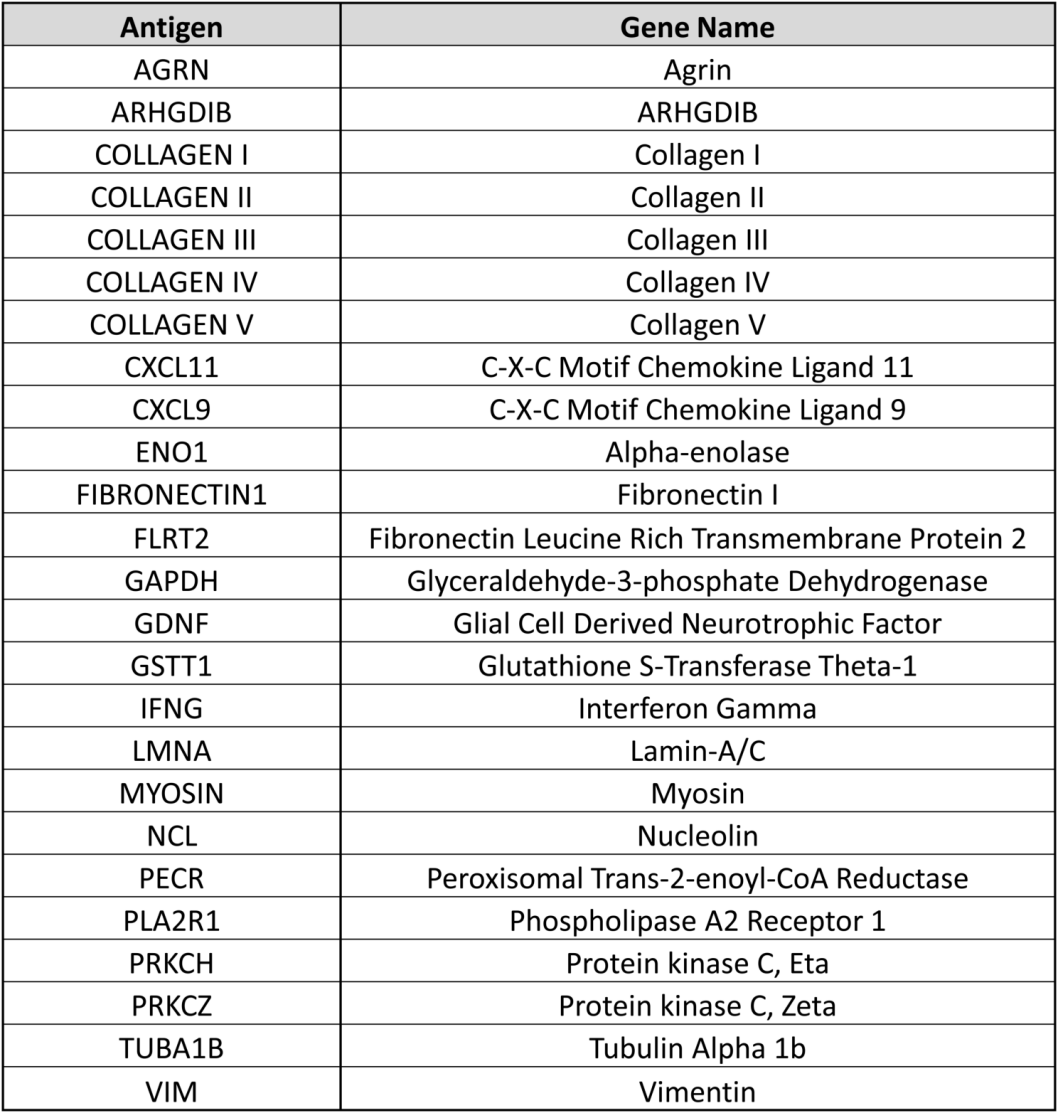
Antigen and gene names are provided for 25 beads representing non-HLA antigenic targets that are shared across the 2 commercially available non-HLA antibody testing panels.

LIFECODES® Non-HLA Antibody Kit (Werfen panel 1) and the LABScreen™ Autoantibody panels (One Lambda Inc. Panel 2), are run using the Luminex xMAP technology. Both assays require use of serum collected from tubes containing no anticoagulant, are untreated and undiluted, and are freshly collected or with limited freeze thaw cycles. A centrifugation step is required to remove aggregates in the serum prior to testing. EDTA, DTT or heat inactivation treatments are not recommended as they may increase background. Panel 1 includes 60 non-HLA targets in one kit. Panel 2 consists of 3 separate kits, allows detection of antibody against 39 non-HLA targets. Both panels include a negative control reagent and a positive control reagent. The positive and negative controls consist of a blend of sera obtained from individuals known to either react or have demonstrated absence of reactivity against most of the non-HLA targets represented on the panels. The MFI cutoffs were determined for each target by the manufacturers. The panels have 25 targets in common (Figure 1).

The non-HLA antibody panels described above were recently used in a retrospective kidney transplant study, in which pre-transplant and post-transplant sera from 12 kidney transplant recipients with early rejection (within 1 month after transplantation) and 18 transplant recipients with no rejection within 3 months were tested (28). The cohort was described as first transplant recipients, unsensitized against HLA antibodies, matched for HLA-A, B and DR antigens, primarily male and white with a median dialysis time of 26 months. The authors found no correlation between the 2 panels. Panel 2 had a higher frequency of positive non-HLA antibody compared to panel 1, and there was no correlation with post-transplant rejection and non-HLA antibody detected using panel 2. A larger retrospective kidney transplant study by Bogdan Obrișcă et al. (manuscript in press), compared reactivity against the 25 shared targets. The study included subgroups with HLA-DSA positive AMR (*n* = 29), HLA-DSA negative AMR (*n* = 28) and recipients with no AMR (*n* = 30). Pre- and post-transplant sera were tested. Using the defined cutoffs set by each vendor, they found that the number of patients with positive antibodies detected by both assays was low. Overall, there was significant heterogeneity between the results of the 2 assays when considering the number of positive non-HLA antibodies and the specificity of the targets. Although it is possible that variability in non-HLA antibody pattern across different cohorts may reflect *in-vivo* tissue specific damage or genetic differences across populations (29), this does not explain the heterogeneity observed in these 2 studies where the same samples were tested with both panels.

### Identifying the appropriate non-HLA target for functional antibodies

**Figure 2:**
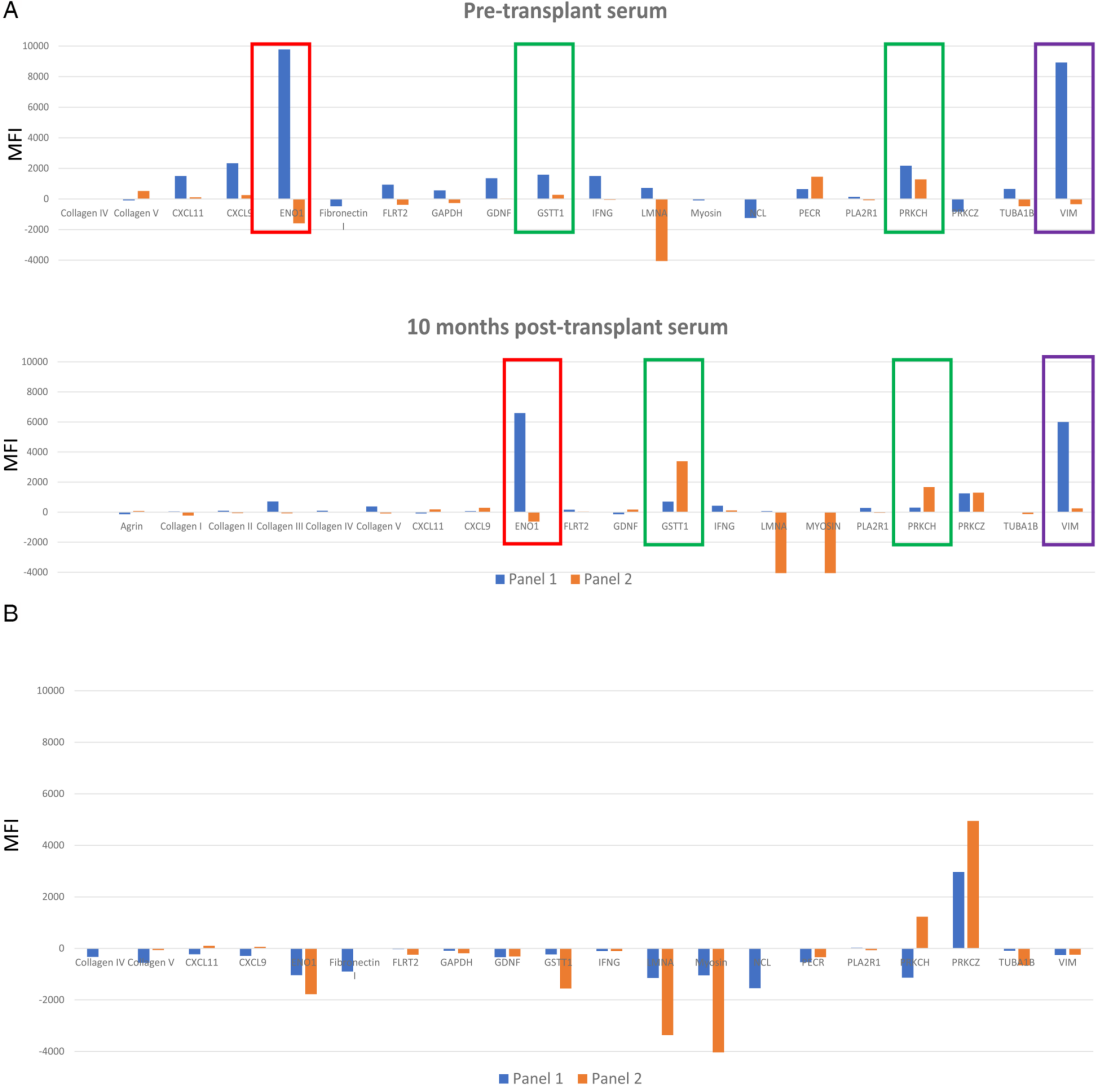
**(A)** A serum sample collected prior to transplantation, and one collected 10 months post-transplantation were tested on both nonHLA antibody panels. Only targets that were positive for at least one nonHLA antibody are represented. Data from panel 1 is displayed with blue bars. Data from non-HLA antibody panel 2 is displayed with orange bars. ENO1, GSTT1, PRKCH and VIM are highlighted to illustrate different results (positive versus negative) between the 2 panels (blue versus orange bars). **(B)** Comparison between 2 panels with serum aged matched non transplanted healthy control. Data from panel 1 is displayed with blue bars. Data from non-HLA antibody panel 2 is displayed with orange bars.

Non-HLA antibodies have been found to be primarily directed against antigenic targets located on the vascular endothelium and are accessible for interaction with an antibody (5). Some non-HLA antibodies are defined as autoantibodies. They are directed against proteins that are expressed in the host as well as a transplanted donor organ and are common in autoimmune diseases (30). These autoantibodies may also develop after injury, when new epitopes from self-antigens become exposed to host immune system or if a self-antigen undergoes post-translational modifications (31, 32). Antibodies against alpha-enolase 1 (ENO1) and vimentin (VIM) are examples of autoantibodies that can be detected using both Luminex non-HLA antibody panels. Genetic differences between organ donors and recipients, outside of the HLA system, could also be targets for an alloimmune response (33). Antibodies against glutathione S-transferase theta class I (GSTT1) is one example. Figure 2 illustrates differences in reactivity against these targets for a sample case discussed below.

### Sample case: comparison of non-HLA antibodies identified with both luminex panels

A 28-year-old male presented with AMR grade 1(I+) with positive C4d staining on biopsy one year after receiving a heart transplant. At the time of transplantation, the recipient was negative for HLA specific antibodies using 2 single antigen bead assays, and the retrospective flow crossmatch was negative with T and B cells. Four months prior to biopsy, dd-cfDNA (CareDx) changed from <0.04% to 0.19%, a 300% increase. AlloMap score increased from 26 to 33; a 27% increase. The repeat flow crossmatch using a serum sample collected at the time of biopsy showing AMR and frozen donor cells remained negative, although a new HLA-DSA against C17 at 5,000 MFI was detected. The patient was also positive for AT1RAbs (>40 U/ml), 2 months post-transplantation. Pre-transplant and post-transplant sera were evaluated for presence of additional non-HLA antibody using commercial Luminex panels 1 and 2 (Figure 2A). Serum from an age matched healthy 34-year-old male (control) was also tested on the panels (Figure 2B). The following targets were positive with one or both panels with the patient's sera and negative with the control serum: ENO1, GSTT1, PRKCH, VIM. One target, PRKCZ was positive with the patient's post- transplant serum and with the control serum.

### Discordant reactivity against ENO1 antigen

In the case study, antibody against ENO1 was positive in panel 1 and negative in panel 2 with the pre-transplant and post-transplant sera. The reactivity was also absent in the control serum. ENO1 is an enzyme with multiple functions during glycolysis (34) and is upregulated under hypoxic or inflammatory conditions (35). It is expressed in the cytoplasm and on endothelial cells, neutrophils, T and B lymphocytes. Antibody against ENO1 develops following post-translational modifications of alpha-enolase in infectious and autoimmune diseases and is reported to activate the complement cascade (34). Since post-translational modifications such as phosphorylation (36) or citrullination influence peptide immunogenicity (37), the optimal reagent would need to consider these modifications.

### Mixed reactivity against VIM

Anti-VIM antibody was detected in the pre-transplant and post-transplant sera for this case using panel 1 and was negative prior to transplantation but changed to weak positive at time of biopsy-proven rejection on panel 2. Reactivity against VIM was absent in the control. VIM is a cytoskeleton protein involved in cell-cell signaling and proliferation (38). Four important antigenic components were described and may be the targets for antibody development (39). Those include: a citrullinated site, 2 cleavage sites for Caspase 3 and Caspase 8 and a V9 site at the tail end, which is a binding site for a mouse monoclonal antibody. Antibody specifically directed against citrullinated peptides were found to be associated with differing disease phenotypes in rheumatoid arthritis (40). It is possible in this case that antigenic targets differ between the 2 panels.

### Concordant reactivity with antibody against GSTT1

GSTT1 is an enzyme that protect tissue from oxidative damage (41). Null variants of these genes result in the absence of this enzyme, and have been linked to increased incidence of coronary artery disease in Indian (42) and Mexican populations (43). The frequency of the null allele ranges between 17 and 20% (44). Antibody against GSTT1 has been detected in null GSTT1 liver transplant recipients who received an organ from a donor positive for GSTT1 gene (45). Recently GSTT1 antibodies have been associated with increased risk of rejection in kidney (26, 46), heart and lung patients (47). Antibodies against GSTT1 are positive on both panels at both time points for the patient and negative with the control. In this case, genetic testing for donor and recipient to determine presence of a null allele is needed. Nevertheless, the consistent pattern of reactivity is encouraging. It is noteworthy to mention the significant difference in the strength of reactivity between the 2 panels which may reflect differences in the concentration of the targets applied to the bead or differences in the method used to apply targets on the beads.

### Treatment approach and outcome

At this time, therapeutics used to treat AMR are employed indiscriminately for non-HLA and HLA antibodies (48). The case patient was treated with pulse steroids and anti-thymoglobulin for hemodynamically significant rejection and then received plasmapheresis and intravenous immunoglobulin, eculizumab (monoclonal antibody directed against complement protein C5). Cardiac allograft function improved with left ventricular ejection fraction 45% after treatment compared to 30% at the time of acute rejection. However, HLA-DSA against C17 remained detectable with unchanged MFI 4,000-7,000 range, and surveillance biopsy at 2 and 4 months after initial presentation remained positive for AMR 1(I+). Additional AMR directed therapy with daratumumab (monoclinal antibody against CD38 on plasma cells and T and B lymphocytes) was provided. Unfortunately, resolution of rejection or effect of daratumumab on HLA-DSA or AT1RAb could not be confirmed as he again presented with acute rejection in the setting of a gap in immunosuppression regimen.

## Conclusion and next steps

Lessons from the evolution of HLA antibody testing have shown that antibodies recognize polymorphic, non-self, antigenic amino acid residues, defined as epitopes and eplets that are accessible to an antibody. Some epitopes are further defined, based on their amino acid sequence, as immunogenic while other epitopes may not elicit an immune response. Additionally, non-polymorphic proteins can trigger immune reactions after post-translational modification, or intracellular targets may become exposed to the host immune system under stress or injurious circumstances (31). Therefore, distinguishing the targets, and replicating their conformational structures as they would appear on the cell surface, is the challenge involved in designing informative assays. Elegant studies like those performed by Duska Dragun, that map the functional AT1RAbs epitopes (20) are needed for every non-HLA antigen described as associated with rejection.

Several non-HLA antigens have been discovered using protein microarrays (49–51),containing thousands of full-length human proteins. Most of the arrays have focused on proteins expressed in the tissues of interest or on the endothelium. These proteins have been immobilized on a glass slide, expressed in a baculovirus system and purified from insect cells (49) or are recombinant human proteins expressed as N- terminal GST fusion proteins (50). Genomic sequence is translated into functional proteins by commonly used vectors (yeast or *E.coli*, insect systems) therefore the expression of the protein is dependent on the similarities with the mammalian processing machinery (52) and certain post-translation modifications such as citrullination may not be present. Furthermore, the functional structure for certain proteins, such as membrane bound proteins and especially those that span the membrane multiple times (like G-protein coupled receptors AT1R and ETAR) (52), may not be preserved. Other considerations include the method selected for immobilizing the protein (surface used and immobilization strategy) which may affect its functionality. These challenges must be considered when designing solid phase assays for detection of non-HLA antibodies.

A recent study used IgG affinity chromatography columns to identify antibodies against donor specific non-HLA antigens in heart transplant recipients with and without AMR (32). Interestingly in this study, acute AMR cases had mainly antibodies directed against extracellular proteins and 86% of these extracellular proteins had known natural polymorphisms (32). Conversely, stable patients and those at one year after the acute AMR episode, had antibodies directed against intracellular proteins, exposed to the host immune system due to loss of cellular integrity caused by damage to the allograft from ischemia reperfusion, viral infection, or another inflammatory process. The antibodies against intracellular non-HLA antigens did not seem to be the primary mediators of graft dysfunction. Based on this study, one may presume that antibodies against extracellular proteins, most of which could be polymorphic, may represent an alloimmune response that initiates the acute AMR, while the consequent cell damage could trigger the development of antibodies against intracellular proteins, prolonging the rejection episode and driving chronic AMR. This study also highlights the importance of organ specific targets. Some targets may only (or mainly) be expressed in certain organs and the same non-HLA antibody may have a different mechanism of action depending on the organ.

Few studies have been done on animal models to further demonstrate the pathogenicity of non-HLA antibodies. In a mice ischemia/reperfusion kidney transplant models the passive transfer of IgG antibodies against Perlecan LG3 fragment led to enhanced dysfunction and microvascular injury compared with passive transfer with control IgG (53). Another study using miniature swine immunized with cardiac myosin demonstrated that immune reactions against myosin where implicated in heart transplant rejection (54). A return to basic science may be needed to better define the pathogenic effects of certain non-HLA antibodies.

The relevance of non-HLA antibody has been demonstrated, although efforts to understand their impact is ongoing and hindered primarily because of the lack of reliable assays and development of standardized testing protocols. It is also unclear whether a single non-HLA antibody can impact graft outcome, or whether a combination of several antibodies is needed. Solid phase immunoassays have made a significant impact in histocompatibility testing. HLA specific bead-based assays are used to increase the specificity and speed of the immunologic assessment when considering donor selection. This progress did not come without pain. Experiences with the Luminex single antigen bead assays for HLA antibody detection have shown that recombinant proteins added to bead may display cryptic epitopes and cause false positive or false negative reactions (55, 56). A noncommercial assay that included 14 non-HLA targets was described in detail with the exact sequence for most of the targets provided (57) and the method used to couple the proteins to the beads (direct or haplotag). Transparent studies describing the composition of the assays are helpful for the interpretation of results. While the relevance of antibodies against AT1R, ETAR and MICA have been demonstrated in the literature, neither one of the commercial Luminex kits includes these targets. Testing that includes these targets would further help with data comparison and validation of a non-HLA antibody panel. We advocate for the continued collaboration between research scientists, transplant immunologists, clinicians, and manufacturers to develop reliable and standardized non-HLA assays that can be used in clinical settings to study the role of non-HLA antibodies in solid organ transplantation. Once appropriate reagents/ tests have been produced, establishing testing parameters at different timepoints may be necessary.

## Data Availability

The original contributions presented in the study are included in the article/Supplementary Material, further inquiries can be directed to the corresponding author.
